# Molecular Identification and Genetic Characterization of Early-Stage Multiple Primary Lung Cancer by Large-Panel Next-Generation Sequencing Analysis

**DOI:** 10.3389/fonc.2021.653988

**Published:** 2021-05-24

**Authors:** Guotian Pei, Mingwei Li, Xianjun Min, Qiang Liu, Dasheng Li, Yingshun Yang, Shuai Wang, Xiaoyu Wang, Huina Wang, Huanqing Cheng, Shanbo Cao, Yuqing Huang

**Affiliations:** ^1^ Department of Thoracic Surgery, Beijing Haidian Hospital, Beijing, China; ^2^ Department of Medicine, Acornmed Biotechnology Co., Ltd, Beijing, China; ^3^ Department of Radiology, Beijing Haidian Hospital, Beijing, China

**Keywords:** early-stage multiple primary lung cancer, multigene sequencing, molecular classification, genetic characterization, epidermal growth factor receptor (EGFR), *L858R*, clonal relationships

## Abstract

**Objective:**

The incidence of early stage multiple primary lung cancer (MPLC) has been increasing in recent years, while the ideal strategy for its diagnosis and treatment remains controversial. The present study conducted genomic analysis to identify a new molecular classification method for accurately predicting the diagnosis and therapy for patients with early stage MPLC.

**Methods:**

A total of 240 tissue samples from 203 patients with multiple-non-small-cell lung cancers (NSCLCs) (n = 30), early stage single-NSCLC (Group A, n = 94), and advanced-stage NSCLC (Group B, n = 79) were subjected to targeted multigene panel sequencing.

**Results:**

Thirty patients for whom next-generation sequencing was performed on >1 tumor were identified, yielding 45 tumor pairs. The frequencies of *EGFR, TP53, RBM10, ERBB2*, and *CDKN2A* mutations exhibited significant differences between early and advanced-stage NSCLCs. The prevalence of the *EGFR L858R* mutation in early stage NSCLC was remarkably higher than that in advanced-stage NSCLC (*P* = 0.047). The molecular method classified tumor pairs into 26 definite MPLC tumors and four intrapulmonary metastasis (IM) tumors. A high rate of discordance in driver genetic alterations was found in the different tumor lesions of MPLC patients. The prospective Martini histologic prediction of MPLC was discordant with the molecular method for three patients (16.7%), particularly in the prediction of IM (91.7% discordant).

**Conclusions:**

Comprehensive molecular evaluation allows the unambiguous delineation of clonal relationships among tumors. In comparison, the Martini and Melamed criteria have notable limitations in the recognition of IM. Our results support the adoption of a large panel to supplement histology for strongly discriminating NSCLC clonal relationships in clinical practice.

## Introduction

Multiple primary lung cancer (MPLC) refers to the synchronous or metachronous occurrence of two or more primary malignant tumors in the lungs of an individual patient and can be further divided into synchronous MPLC (sMPLC) and metachronous MPLC (mMPLC), the latter of which is defined by a diagnosis interval of 6 months between tumors (1). In 1924, Beyreuther first described cases of “double primary lung cancer” and introduced the concept of MPLC (2). MPLC is believed to be a rare disease. However, recent clinical evidence has shown that the incidence of MPLC has been increasing, which may be attributed to advances in chest computed tomography (CT) and increased awareness among clinicians regarding MPLC screening (3). Therefore, higher-accuracy diagnostic methods and better treatment options for MPLC are urgently needed. Multinodular lesions are usually observed in approximately 16% of patients with operable stage I, II, and III non-small-cell lung cancer (NSCLC) by preoperative imaging analysis (4). Overall, MPLC accounts for 1–8% of all multinodular lesions according to a recent report (5), and adenocarcinoma accounts for 86.5% of multinodular lesions, which may be related to the higher incidence of lung adenocarcinoma (6). Chang et al. reported that the upper lobes of both lungs are prone to MPLC, and multiple lesions have the same pathological type in 50–70% of patients (7).

In 1975, Martini and Melamed proposed some criteria for differentiating multiple primary lung tumors from pulmonary metastatic tumors (8). However, this empirical classification does not include molecular analysis and cannot fully identify the link between multiple tumors. The histological characteristics of multiple tumors often overlap in lung cancer, especially in adenocarcinoma (9). Therefore, it is challenging to distinguish multiple primary tumors and multiple intrapulmonary metastases in the absence of molecular characteristics. Currently, intratumor heterogeneity is often interpreted using the trunk-branch model (10). In this model, trunk gene mutations drive tumor growth in each subcloning and tumor region. As the disease progresses, branch gene mutations occur heterogeneously in primary lesions and/or metastases and may induce intratumor heterogeneity. Based on this theory, lesions with multiple identical mutations could originate from the same clone. Numerous studies have shown that mutations in certain proto-oncogenes and cancer suppressor genes, such as *EGFR*, *KRAS*, and *BRAF*, can be used as molecular markers in multiple lung cancers (11, 12). However, only a few hotspot cancer driver gene mutations have been analyzed, and these mutation hotspots are not sufficiently reliable to analyze the differentiation of MPLC and intrapulmonary metastasis (IM).

Recently, numerous studies thoroughly investigated the genomic changes and clonal structures of advanced lung tumors (13, 14). However, there are relatively few reports regarding the genomic characteristics of early stage NSCLC, especially the molecular clonal relationship among tumors in patients thus far. Therefore, we adopted next-generation sequencing (NGS) to detect multiple cancer-related genes in multiple lung cancer (MLC) using tumor samples obtained *via* surgical resection and compared the identification-based molecular mutation spectrum with the histopathological evaluation of the tumors. Importantly, the genomic characterizations of early stage MPLC were comprehensively defined by comparing early stage NSCLC and advanced-stage NSCLC.

## Materials and Methods

### Patient Enrolment

**Figure 1:**
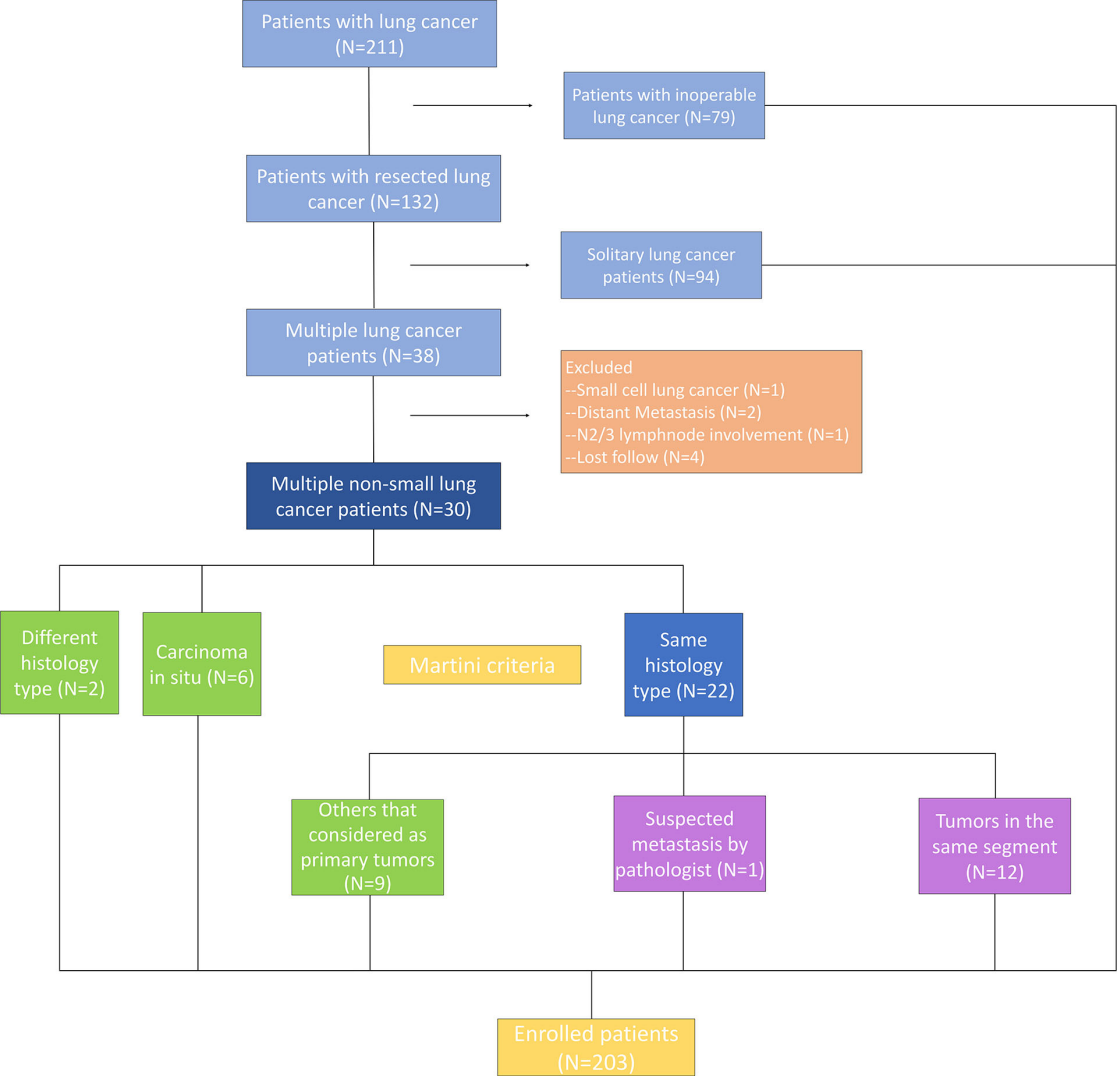
The flowchart of the patients’ inclusion criteria.

The subjects were patients with multiple NSCLC who underwent surgical resection synchronously or metachronously at the Department of Thoracic Surgery of Beijing Haidian Hospital between September 2017 and December 2019. Patients who received neoadjuvant therapy and had extrathoracic metastases were excluded (**Figure 1**). A total of 67 surgical specimens sufficient for histological and molecular analyses were obtained from 30 patients who had more than one tumor and were eligible for selection in this study. Synchronous tumors, or metachronous tumors, were defined by a diagnosis interval of 6 months or less. All patients underwent a chest CT scan before the surgery. Meanwhile, 94 patients who underwent surgical resection with early stage (IA stage) disease were enrolled in Group A, and 79 specimens were obtained using ultrasound-guided transbronchial needle aspiration from 79 patients who had advanced lung cancer and were allocated to Group B. The study was approved by the Medical Ethics Committee of Beijing Haidian Hospital (No. 2020-041), and individual consent for this retrospective analysis was waived.

### Criteria of Martini and Melamed

According to the Martini and Melamed criteria published in 1975 (8), multiple NSCLCs are classified into MPLC by histological type and clinical data. If they presented with similar histological types, different anatomical distributions, different origins of carcinoma *in situ*, long intervals, and no lymphatic or systemic metastasis in different segments, synchronous tumors are classified as MPLC. If the diagnosis interval was more than two years, metachronous tumors are classified as MPLC. Histologic assessment of tumor relatedness was performed by experienced thoracic pathologists.

### Targeted Multigene Panel Sequencing

For each tumor, DNA was extracted from the formalin-fixed paraffin-embedded block containing the highest percentage of tumor cells. DNA extraction and NGS analysis were performed by using an Acornmed panel targeting 808 cancer-related hotspot genes that provided data on non-synonymous somatic mutations, copy number alterations, small insertions or deletions, copy number variants and rearrangements. This analysis focused on targetable genetic alterations annotated by categories of evidence Levels 1–3 and Level R1 in OncoKB (Memorial Sloan Kettering Cancer Center, New York, NY, http://oncokb.org/). Synonymous mutations are detected and maintained in the database but not clinically reported.

Tissue DNA was extracted using an QIAamp Genomic DNA Kit (Qiagen GmbH). Quality and quantification of the DNA were measured using an Agilent 2100 BioAnalyzer (Agilent Technologies, Inc.) and a Qubit ds DNA HS detection kit (Thermo Fisher Scientific, Inc.). Various libraries were hybridized with the 808-gene panel that contained coding regions and introns. The target-enriched libraries were pooled and sequenced on an Illumina HiSeq2500 NGS platform. The quality criteria used as endpoints were a detection threshold of 5% and an average coverage depth of 10,000×. The genome data were processed with the relevant bioinformatics platform to identify multiple types of gene mutations. By analysing somatic mutations, including coding base substitution and fragment insertion and deletion, the tumor mutational burden (TMB) was estimated as the number of mutations per million bases.

### Statistical Analyses

GraphPad Prism and SPSS were used for statistical analysis. Differences in continuous variables were assessed using unpaired t-tests. Fisher’s exact test or χ2 test was used to analyze the association of clinical characteristics, genetic characteristics, and molecular markers of immunotherapy between different groups. A two-sided *P <*0.05 was considered to be statistically significant.

## Results

### Clinical Characteristics of Patients With MLC

We identified a total of thirty patients with NGS performed on >1 resected NSCLC tumor. The proportions of females, non-smokers, and patients with adenocarcinomas were 71.3, 80.0, and 100%, respectively. The median age was 60 years (range, 46–82 years). For 22 patients (73.3%), all the tumors were located on the same side. Twenty-four patients had two tumors, five patients had three tumors, and one patient had four tumors, for a total of 67 individual tumors. Most of the tumors were detected at early stages, including 25 foci (37.3%) at stage IA1. The maximum diameter of 34 tumors was ≤10 millimetres (50.8%). Invasive adenocarcinoma (IAC) (n = 34) and minimally invasive adenocarcinoma (MIA) (n = 21) were the main pathological types. Imaging examination showed that there were 16 tumors with pure ground-glass opacity (GGO) and 30 tumors with mixed-density ground-glass nodules (GGNs) (**Table 1**). Clinically, either all patients were considered to have separate primary tumors or the relationship of the tumors was uncertain at the time of surgery; none of the patients was known to have IM prior to surgery.

**Table 1:** Clinical and radiological characteristics of 30 patients with MLC.

Patient characteristics (N = 30)	Number (%)
Sex, n (%)	
Male	8 (28.7%)
Female	22 (71.3%)
Age (year), y (range)	
Median	60
Range	41–78
≤60	15 (50.0%)
>60	15 (50.0%)
Smoking history, n (%)	
Yes	6 (20.0%)
No	24 (80.0%)
Tumor chronology, n (%)	
Synchronous	25 (83.3%)
Metachronous	5 (16.7%)
Tumor distribution, n (%)	
Ipsilateral (same lobe)	11 (36.7%)
Ipsilateral (different lobe)	11 (36.7%)
Contralateral	8 (26.7%)
Tumor characteristics (n = 67)	
Stage*, n (%)	
AAH	1 (1.5%)
0	5 (7.46%)
IA1	25 (37.3%)
IA2	15 (22.4%)
IA3	6 (9.0%)
IB	1 (1.5%)
IIB	12 (17.9%)
IV	2 (3.0%)
Histology, n (%)	
AAH	1 (1.5%)
AIS	5 (7.5%)
MIA	21 (31.3%)
IAC	34 (50.7%)
SCC	3 (4.5%)
MA	3 (4.5%)
Side, n (%)	
Left	22 (32.8%)
Right	45 (67.2%)
Maximum diameter, mm (range)	
Median	9.4
Range	2.5–40
≤6	14 (20.9%)
6-10	20 (29.9%)
10-20	22 (32.8%)
>20	11 (16.4%)
Radiological feature	
Solid	20 (29.9%)
Subsolid	30 (44.8%)
pGGO	16 (23.9%)
Thin-walled cavity	1 (1.5%)

*At primary surgery according to the International Union Against Cancer (UICC) eighth TNM staging.

MLC, multiple lung cancer; AAH, atypical adenomatous hyperplasia; AIS, adenocarcinoma in situ; MIA, minimally invasive adenocarcinoma; IAC, invasive adenocarcinoma; SCC, squamous-cell carcinoma; MA, Mucinous adenocarcinoma; pGGO, pure ground-glass opacity.

### Tumor Molecular Characteristics

**Figure 2:**
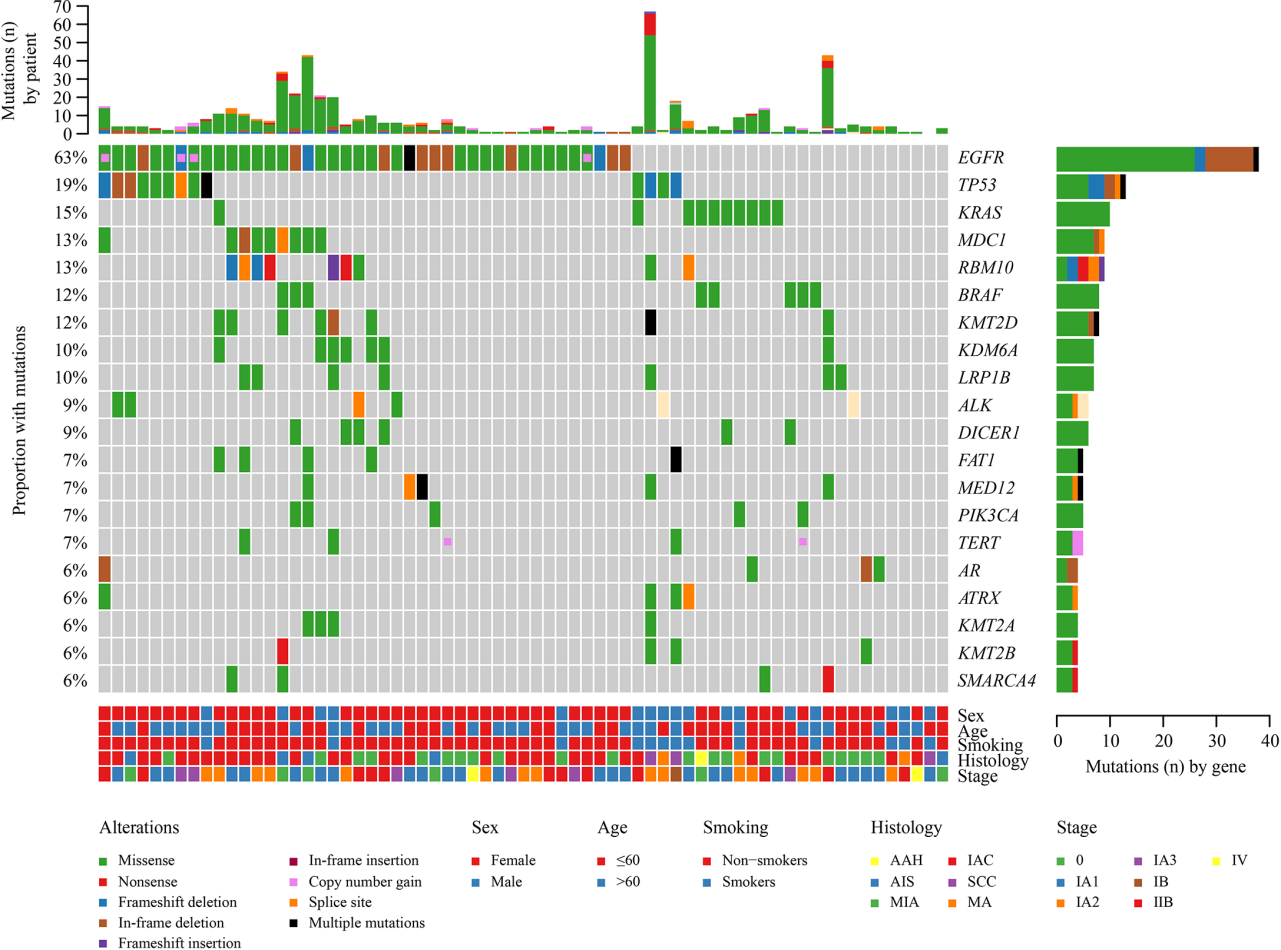
Landscape of genomic alterations in 67 multiple lung cancer samples. Genetic mutations were identified by targeted next-generation sequencing in the tumor tissues of the patients. The upper panel shows the numbers of non-synonymous single-nucleotide variants, small insertions or deletions, and copy number variants in each tumor. The heat map below shows the genes with somatic mutations sorted according to the mutation frequency. Clinical features are annotated in the lower panel.

Overall, a total of 542 mutations were detected in these 67 tumors. Major oncogenic driver alterations were identified from 59 out of 67 (88.06%) tumors, and at least two mutations were detected in 80.6% (54/67) of specimens. The most commonly mutated genes were *EGFR* (63%, including 23 patients with *EGFR L858R*, 12 with exon 19 deletions, and seven with rare mutations), *TP53* (18%), *KRAS* (15%), *RBM10* (13%), *MDC1* (13%), *BRAF* (12%), and *KMT2D* (12%) (**Figure 2**). Moreover, *ALK* fusion was observed in two patients, and *ROS1* fusion was seen in one patient. Only one lesion had two concomitant driver mutations. Gene amplification (*e.g*., *EGFR, TERT, MYC*, and *ERBB2*) was identified in tumor samples from 10 patients but was present only in paired tumors for one patient (**Figure 2**).

### Comparison of Genomic Characterization Among Early Stage and Advanced-Stage NSCLC

Since most MPLCs are early stage, the molecular characteristics of early stage lung cancer help identify the clonal relationship between different primary tumors. To comprehensively investigate the genomic characterization of stage IA lung cancer, 94 patients (Group A) with early stage NSCLC and 79 patients with advanced-stage NSCLC (Group B) were included in the study. A comparative analysis of the two groups was further performed. The clinical characteristics of Groups A and B are shown in **Tables S1 **and** S2**, respectively. Moreover, sex, smoking history, and pathological type were significantly different between Groups A and B (**Table S3**).

**Figure 3:**
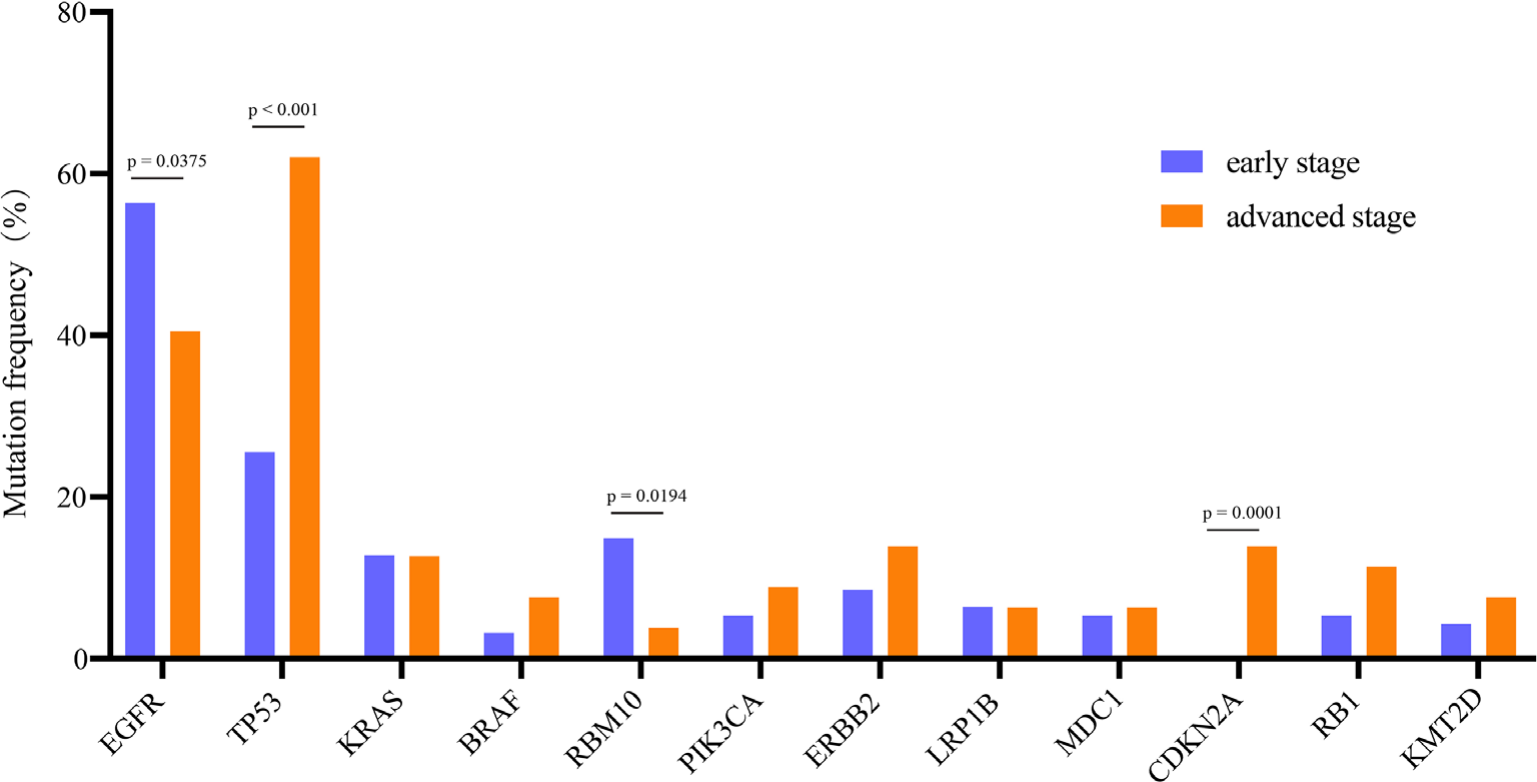
Comparison of the prevalence of frequently mutated genes in early stage and advanced-stage NSCLCs. The commonly mutated genes are arranged in order on the horizontal axis. The vertical axis represents the mutation frequency obtained from a different cohort.

**Figure 4:**
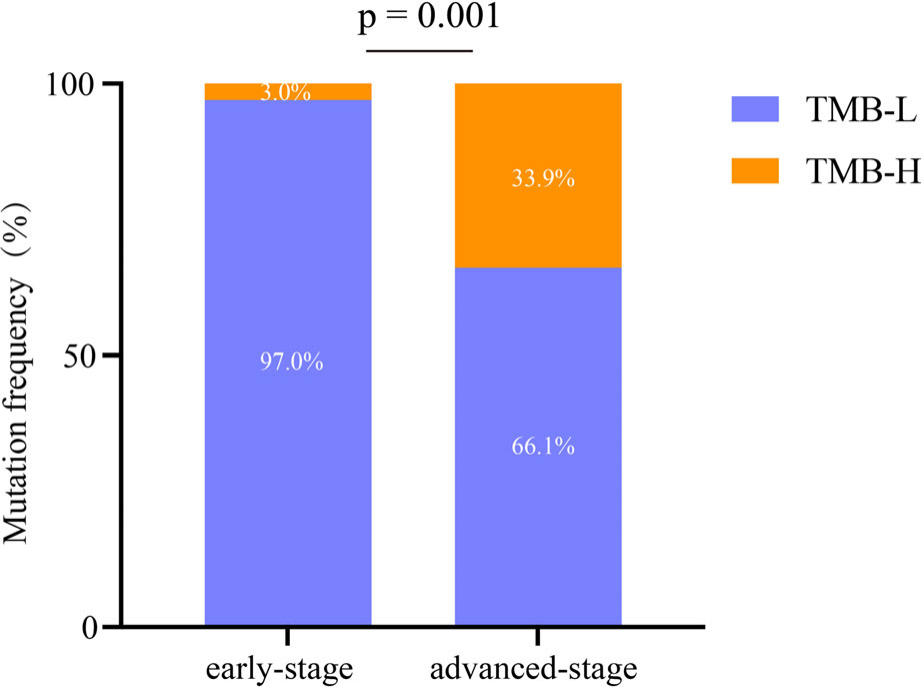
Analysis of the characteristics of immunotherapy biomarkers in early stage and advanced-stage NSCLCs. TMB-H, high tumor mutation burden; TMB-L, low tumor mutation burden.

A total of 520 genomic mutations were identified in Group A. Frequently mutated genes included *EGFR* (56%), *TP53* (26%), *RBM10* (15%), *KRAS* (13%), and *KMT2C* (10%) (**Figure S1**). In Group B, a total of 873 genetic mutations were identified. *TP53* (62%) was the most commonly mutated gene, followed by *EGFR* (41%), *CDKN2A* (14%), *ERBB2* (14%), *KRAS* (13%), and *RB1* (11%). Among all the mutations, *ALK* fusion was observed in four patients, *ROS1* fusion in one patient, and *RET* fusion in two patients (**Figure S2**). Compared with those in advanced-stage NSCLC, significantly more genomic mutations in *EGFR* and *RBM10* (*P* = 0.038 and *P* = 0.019, respectively) and significantly fewer mutations in *TP53* and *CDKN2A* (*P* < 0.0001 and *P* = 0.0001, respectively) were identified in early stage MPLC (**Figure 3**). Furthermore, the TMB was evaluated among the patients based on the mutation data. Advanced-stage NSCLC showed a higher frequency of a high TMB than early stage NSCLC (*P* = 0.001) (**Figure 4**). According to the results, early stage NSCLC tended to exhibit a low TMB more often than advanced-stage NSCLC.

### Comprehensive Analysis of *EGFR* Alterations

**Figure 5:**
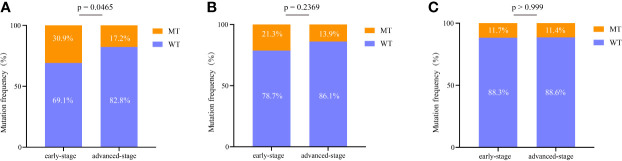
Comprehensive analysis of *EGFR* mutations in early stage and advanced-stage NSCLC. **(A)** Comparison of the difference in the *EGFR L858R* mutation between the two groups. **(B)** Comparison of the difference in the *EGFR exon 19 deletion* between the two groups. **(C)** Comparison of the difference in the other *EGFR* mutations (excluding *EGFR L858R* substitution and *exon 19 deletion*) between the two groups. 19del, exon 19 deletion; WT, wild type; MT, mutation type.

*EGFR* is the most commonly mutated gene in early lung cancer, and thus we further analyzed its subtypes. Among all *EGFR* mutations, the most common were *EGFR L858R* substitution and *exon 19 deletion* (*19Del*). The frequencies of different *EGFR* mutation types were compared between Groups A and B. For the *EGFR L858R* mutation, a remarkable difference between Groups A and B was identified (*P* = 0.047) (**Figure 5A**). However, for *EGFR 19Del*, no significant difference was observed between the two groups (*P* = 0.2369) (**Figure 5B**). Additionally, for other *EGFR* mutations (excluding *EGFR L858R* substitution and *19Del*), no striking difference between Groups A and B was observed (**Figure 5C**).

### Clonality Assessment Based on Large Panel NGS Results

To determine the clonal relationship between two or more tumors, we compared somatic mutations and copy number alterations. **Table 2** summarizes the NGS-classified tumors among the patients as detailed below. Twenty-one patients (70%) exhibited inconsistent driver mutations and entirely unique mutation profiles in each tumor (**Figure S3**). These cases were classified as definite MPLC. Conversely, three patients (10%) shared driver mutations and additional multiple (≥2) non-synonymous somatic alterations (mean 5.3, up to 10). These cases were thus classified as definite IM. Compared with the number of shared mutations, the number of unique mutations in IM was substantially lower.

**Table 2:** Patients with MLC classified by the Martini and Melamed criteria and molecular methods.

Case No.	Martini & Melamed criteria	Mutational evaluation	Number of matching mutations	Matching genes
Martini and Melamed criteria-based MPLC cases (n = 18)				
1	MPLC	MPLC	0	–
2	MPLC	MPLC	0	–
3	MPLC	MPLC	0	–
6	MPLC	MPLC	0	–
7	MPLC	MPLC	0	–
10	MPLC	MPLC	0	–
12	MPLC	MPLC	1	*TERT Amplification*
13	MPLC	MPLC	0	–
15	MPLC	MPLC	0	–
20	MPLC	MPLC	1	*EGFR L858R*
21	MPLC	MPLC	0	–
24	MPLC	MPLC	0	–
25	MPLC	MPLC	0	–
26	MPLC	MPLC	0	–
27	MPLC	MPLC	0	–
9	MPLC	IM	4	*EGFR/ALK/TP53/IKZF2*
29^a^	MPLC	IM	1	*EGFR L858R/T790M*
30	MPLC	IM	1	*EGFR 19Del*
Martini & Melamed criteria-based IM cases (n = 12)				
4	IM	MPLC	0	–
5	IM	MPLC	0	–
11	IM	MPLC	0	–
14^a^	IM	MPLC	0	–
16	IM	MPLC	1	*EGFR L858R*
17^a^	IM	MPLC	1	*EGFR L858R*
18	IM	MPLC	0	–
19	IM	MPLC	0	–
22	IM	MPLC	0	–
23	IM	MPLC	1	*EGFR L858R*
28	IM	MPLC	0	–
8	IM	IM	10	*EGFR/KDM6A/KMT2D*

a Tumor 1 vs Tumor 2.

IM, intrapulmonary metastasis; MPLC, multiple primary lung cancer.

A total of seven patients (20%) shared single identical mutations (**Figures S4A, B**); their classification was adjudicated individually by extended molecular review. Six patients shared single *EGFR* hotspot mutations (*L858R* in five and *19Del* in one), and one patient shares an other/rare mutation (*TERT Amplification*); each tumor also harbored an abundance of unique mutations, ranging from four to 28 mutations per tumor, with no shared additional mutations.

Of those, five patients shared a single *EGFR L858R* driver mutation. Classification of those tumors as MPLC was supported by 1) the fair probability of coincidentally shared driver mutation; in particular, given the prevalence of *EGFR L858R* mutation in our population of 52%, the odds of coincidental occurrence of this mutation in two unrelated tumors was very high; and 2) the substantially higher unique/total mutation ratio (>75%) compared with definite IM in our series.

One other patient shared a single *TERT Amplification*. These tumors also harboured distinct *EGFR 19Del versus BRAF V601E* driver mutations plus multiple unique non-synonymous mutations in each tumor, resulting in a high-probability classification of MPLC with coincidental *TERT Amplification*.

Lastly, one patient shared a single *EGFR 19Del* mutation with one and five unique mutations per tumor. On manual review, all mutations had low VAF (<10%). Such findings indicated low tumor purity and the likelihood of the incomplete detection of mutations. Moreover, there was no significant difference in *EGFR 19Del* in early and advanced-stage NSCLC; thus, the tumor was classified as an unambiguous IM.

**Figure 6:**
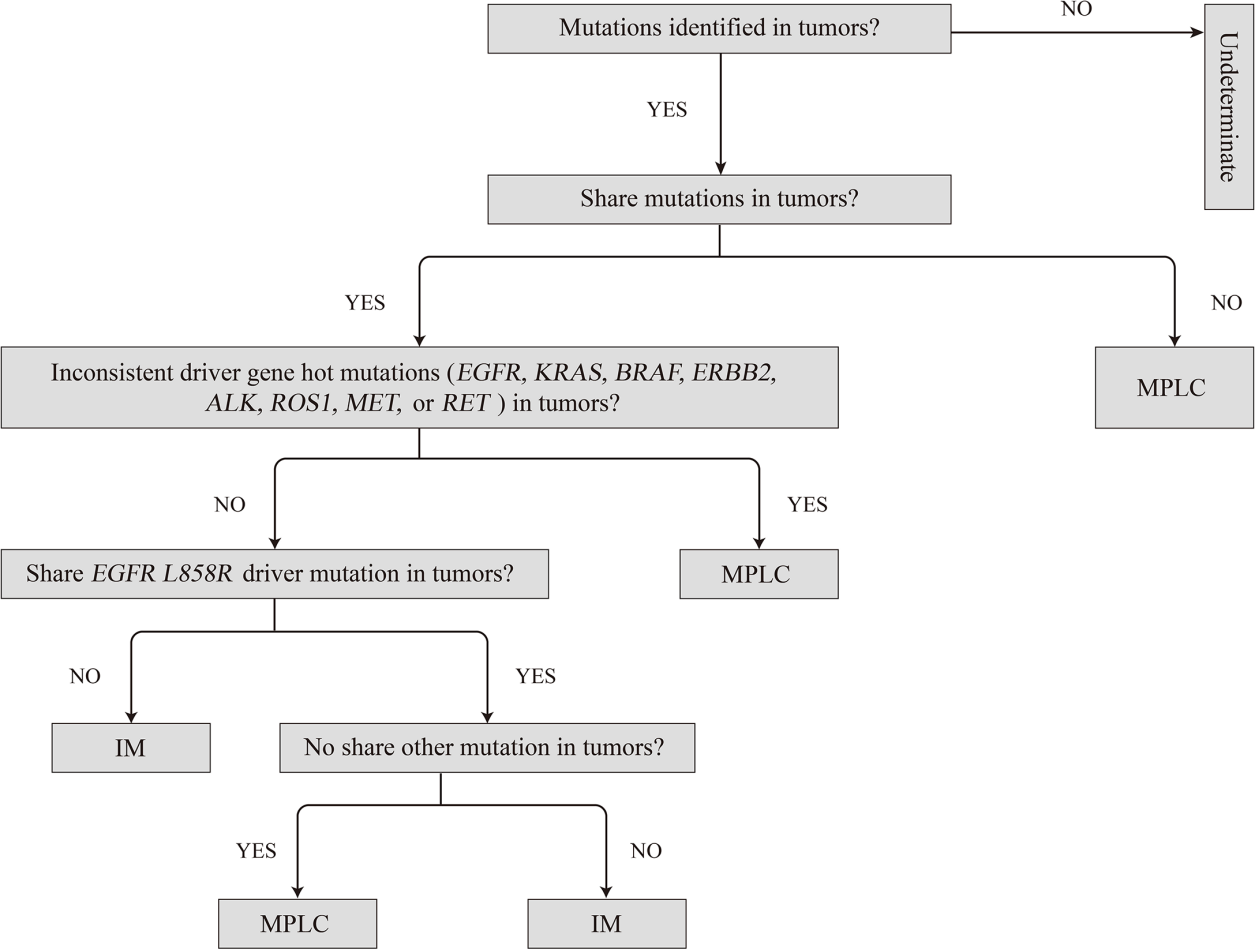
Proposed algorithm for classifying multiple primary lung cancers based on molecular criteria. IM, intrapulmonary metastasis; MPLC, multiple primary lung cancer.

Therefore, we propose herein a molecular method for classifying patients as having MPLC or IM as *per* the analysis of multiple cancer-related gene somatic mutations and the molecular mutation characteristics of MPLC (**Figure 6**), which is described as follows: (1) MPLC can be identified when tumors have no mutation in common or when they had different driver-gene hotspot mutations (*EGFR*, *KRAS*, *BRAF*, *ERBB2*, *ALK*, *ROS1*, *MET*, or *RET*); (2) MPLC can be identified when an *EGFR L858R* mutation is the single consistent mutation between the lung cancer tumors in the patient; (3) IM can be identified when the same driver gene mutation (exclusive to *EGFR L858R*) is shared between tumors or when all alterations are common between the tumors in the patient; and (4) the tumor could not be classified if no mutation is detected in the tumor lesions. Then, the tumor should be classified separately based on the histopathological and clinical data.

### Consistency Between Molecular Methods and Martini Criteria in Identifying MPLC

According to the Martini and Melamed criteria, 13 patients were classified as having MPLC, whereas the remaining patients were classified as having IM. Using the NGS molecular classification, we identified four patients with IM and 26 patients with MPLC (**Table 2**). According to the above results, our molecular method analysis showed 53.3% (16/30) consistency with the clinical and histopathological classification of MPLC. With respect to previous literature on the molecular identification of MPLC, our results showed consistency with the histopathological classification (15–19). However, of the patients histopathologically identified with MPLC (n = 18), fifteen (83.3%) were diagnosed with MPLC by NGS molecular classification as well, and the remaining patients showed matching mutations. Among these, the paired tumors of three patients harbored consistent driver mutations (excluding *EGFR L858R*) and ≥two matching mutations. Of the patients histopathologically identified with IM (n = 12), only one (8.3%) was diagnosed with IM by NGS molecular classification as well. Among these, the paired tumors from eight patients showed no matching mutations, and three patients shared single *EGFR L858R* mutations.

**Figure 7:**
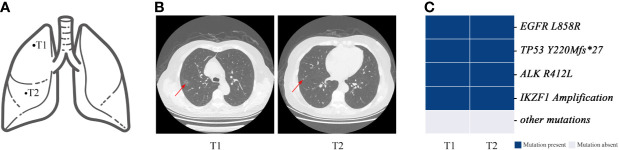
Genomic mutations and computed tomography (CT) images of patient 9. Patient 9 was classified as having MPLC using the Martini and Melamed criteria. The lesions of the patient were found to have multiple consistent mutation sites and were diagnosed as IM by mutation evaluation. **(A)** Schematic diagrams of lung lesions. **(B)** and **(C)** Corresponding CT images and mutation distributions of patient 9.

Notably, the classification results for P9 were inconsistent. According to the imaging results, the two tumors of the patient were 10–20 mm in diameter, and both showed early mixed GGNs with pre-infiltration lesions shown on pathological examination (**Figures 7A, B**). Molecular results showed that the mutations of the two tumors are exactly the same (**Figure 7C**). Although P9 was diagnosed with MPLC based on the histopathological classification, they more likely had intrapulmonary metastases based on the molecular characteristics.

**Figure 8:**
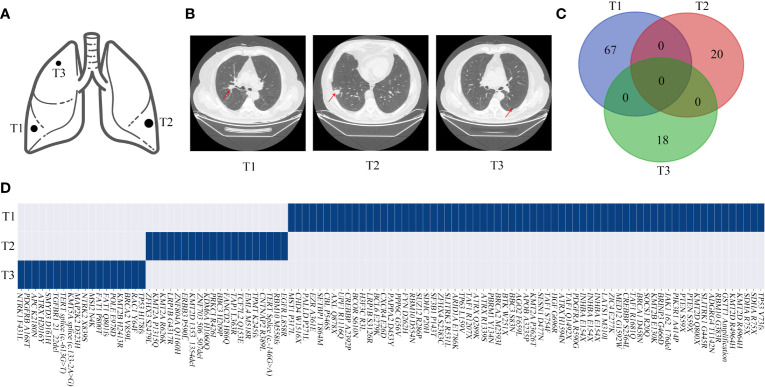
Genomic mutations and computed tomography (CT) images of patient 13. **(A, B)** Schematic diagrams of lung lesions and corresponding CT images. **(C, D)** Relationship of the mutations between different lesions exhibited by a Venn diagram and the mutation distributions of patient 13. The arrow indicates the tumor lesions.

In particular, it is challenging to clinically evaluate the relationship between the tumors of MPLC if they are pathologically classified as squamous cell carcinoma because of a lower frequency of driver mutations. Conventionally, metastasis is often considered by clinicians if the squamous cell carcinoma is pathologically identified in two tissues in one patient, especially with heterochrony. In our study of P13, the first primary tumor (squamous cell carcinoma) was observed in November 2018; the second tumor (adenocarcinoma) with an *EGFR L858R* mutation was observed in March 2019, and the patient was diagnosed with MPLC; the third tumor (squamous cell carcinoma) was observed in October 2019 and had the same pathology as the first tumor (**Figures 8A, B** **)**. The three tumors had inconsistent genetic mutations and may have a primary clonal relationship, suggesting MPLC (**Figures 8C, D** **)**. The third tumor was a metachronous multiple primary lung squamous cell carcinoma. Our study has suggested that molecular methods can assist in the diagnosis of metachronous multiple squamous cell carcinomas.

## Discussion

MPLC has historically been considered a rare phenomenon, but it has been reported with increasing frequency due to improvements in imaging technology and surveillance mechanisms. However, it remains difficult to distinguish the second primary lesion and metastases (20). For clinical management, it is important to classify the disease as intrapulmonary metastasis or multiple primary lung carcinoma to the define TNM classification and optimize the therapeutic options. With the development of high-throughput sequencing technology, molecular genetic analysis using cancer driver gene mutations as biomarkers can greatly assists in distinguishing multiple primary tumors and metastatic tumors in patients with lung cancer (21). Recent evidence has shown that the analysis of genetic mutations in MPLC patients is limited by the small number of cancer driver gene mutations (15, 16). Begg et al. believed that a single or a small number of gene loci are insufficient for identifying IM and MPLC (22). To improve the accuracy of diagnosis, at least 20 gene mutation sites are needed to distinguish IM from MPLC. Multi-gene assessment of MPLC is essential. Moreover, the current knowledge of the molecular characteristics of MPLC is insufficient to provide an accurate molecular diagnosis.

In our study, multigene panel sequencing was used to comprehensively analyze the genomic signature in early stage NSCLC. According to our results, a significant difference in genetic characteristics between early stage and advanced-stage NSCLC was observed. We found that early stage NSCLCs are characterized by a high frequency of driver gene mutations and a small number of mutations. Moreover, *EGFR*, *TP53*, *RBM10*, *LRP1B*, and *MDC1* mutations were observed in early stage NSCLC. These mutations are also found in the early lesions of AIS/MIA (23). Hence, these genes may be involved in early tumorigenesis.

Consistent with previous studies, *EGFR* was the most commonly mutated gene in early stage lung cancer (24). In this study, *EGFR* mutations were identified in 56% of patients with early stage NSCLC, which is higher than that in previous reports (30–40% in patients with early stage lung adenocarcinoma in Asia) (25). In this study, most patients were women without a smoking history. Additionally, previous studies have shown that GGO nodular lung adenocarcinoma had a higher frequency (up to 63%) of *EGFR* mutations than other types of adenocarcinoma (26). Consistently, our study also found that most patient samples had GGO features. Therefore, the specific clinical characteristics of the enrolled patients might correlate with the high prevalence of *EGFR* mutations. Further analysis showed that the prevalence of *EGFR L858R* mutations was significantly different between early and advanced-stage NSCLC, but the frequencies of *EGER 19Del* and other *EGFR* mutations (excluding *EGFR L858R* substitution and *19Del*) were not significantly different. All these results indicate that early stage NSCLC shows distinct *EGFR L858R* mutation characteristics, which may be associated with its carcinogenic properties.

Unlike previously used smaller gene panels (27, 28), large-panel NGS provides a way to examine multiple mutations simultaneously, yielding robust discrimination of tumor relatedness. In our research, an 808-gene panel NGS approach was used to analyze the surgically excised tumor of a patient with MLC. In this series, the tumors harbored a median of 4 (up to 67) non-synonymous somatic alterations per case. Thus, for tumors classified as IMs, multiple shared alterations (median 4, up to 10) and consistent driver mutations were present. We also found that the large-panel NGS robustly identified MPLC by demonstrating entirely unique mutational profiles comprising multiple alterations (median 4, up to 67 per tumor pair), representing an advantage over panels that examine only major drivers or non-comprehensive NGS. In particular, we found that comprehensive NGS allows the clear recognition of MPLC with coincidentally shared single hotspot mutations. Overall, our molecular classification was able to establish definitive tumor clonal relationships in virtually all tumor pairs during the study period.

Recent advances in tumor molecular biology have resulted in the identification of several candidate biomarkers, such as *EGFR*, that can be used in the diagnosis of MPLC (29). Molecular classification is based on the presence of a common driver gene as a biomarker of a similar tumor origin. This assumption may be controversial, particularly when a common driver alteration is used as a unique classifier. We found that MPLC based on mutational evaluation was enriched in *EGFR* mutations (59%), in line with the lack of smoking history, the female sex, and the Asian ethnicity of those patients. Multifocal early stage tumors are frequently present in such patients. Indeed, 5 patients (P16, P17, P20, P23, and P30) consistently presented with the *EGFR L858R* mutation in this study, but previous studies have also reported that *EGFR* driver gene mutations between tumors are consistently judged as MPLC. Thus, mutations at the same hotspot site need to be interpreted more cautiously, especially the *EGFR L858R* mutation. Notably, in European and American populations, MPLC is dominated by *KRAS* mutations (19, 30), likely reflecting the overall known geographic differences in genomic profiles of NSCLC. The only other instance of coincidentally shared hotspot mutations in our series was a *TERT Amplification* in an otherwise unambiguous MPLC. In this study, we illustrated that a large NGS panel can readily identify MPLC despite the presence of shared single hotspot mutations by demonstrating numerous additional unique mutations in each of the tumors. A significant advantage of the comprehensive NGS panel of the type used here is its ability to discriminate MPLCs that share a single common hotspot mutation by chance.

In fact, in our series, shared *EGFR L858R* mutations were almost as likely to occur coincidentally in MPLC as in IM. Notably, there was an identical mutation (including *EGFR L858R* mutation) in the two separated tumor lesions from patient 9 (P9). Many studies have shown that the concordance rate of gene mutations in the primary tumor and metastasis is >90%, while that in multiple lung tumors, *e.g.*, MPLC, is 10.3–32.6% (31, 32). We believe that the tumor pairs of these two patients were metastatically assessed from a molecular perspective. However, CT showed that these two patients had partially solid and pre-invasive tumors without lymphatic metastasis. The results showed that IM might also be found according to the histopathology of MPLC, indicating that they may have aerosol metastasis. Therefore, the 2-year disease-free survival of the patient should be observed.

In our research, the molecular method based on an evaluation of driver gene mutations and the number of alterations for classifying MPLC were summarized, contributing to accurately defining the tumor stage and adjusting the treatment strategies. When comparing the performance of histologic assessment to the definitive NGS molecular classification, we found that histologic prediction was consistent in 53.3% of patients, and up to 47% of tumor stages were changed. Overall, this was similar to the discrepancy rates that ranged from 30 to 50% across different platforms in prior studies (17, 33). Among patients histopathologically diagnosed with MPLC, we found a good concordance rate (83.3%) with the diagnosis based on the mutational evaluation. In contrast, among patients histopathologically diagnosed with IM, the concordance rate with the diagnosis based on the mutational evaluation was only 8.3%. Difficulties with histologic prediction were substantially more frequent in the recognition of IM than MPLC. Thus, there is a limitation in the histopathological diagnosis of MLC, and clonality analysis by mutational evaluation may be helpful for distinguishing MPLC from IM.

This molecular method optimizes the previous methods based on genomic alterations, provides new criteria, and may improve the diagnostic accuracy for early stage MPLC, especially in cases where the lesions have the same pathological type and cannot be identified traditionally (36.7%). We note that the histology in this series was dominated by adenocarcinomas, and only two tumors in a patient were squamous cell carcinomas, precluding detailed analysis of this subset. Nevertheless, these cases illustrate the effectiveness of a large NGS panel in unambiguously establishing tumor relationships in such pairs due to their high tumor mutational burden even in the absence of driver alterations. Conversely, due to the relative homogeneity of cytologic features in squamous cell carcinoma (9), histologic features may not be sufficiently distinctive for the definitive classification of tumor relationships.

Although this molecular method performed well in the diagnosis of early stage MPLC, several limitations of the present study should be acknowledged. The size of our study cohort was relatively small, and further investigation and larger prospective studies are necessary to find more definitive molecular clonal relationships. Since some patients have not reached 2 years after surgery, and the follow-up time span is not long enough to appropriately assess long-term survival. Therefore, the present study has not yet analyzed these data. However, no incidents have occurred in the patients so far. Studies with a larger cohort of patients, long-term follow-up, and survival data would be helpful in substantiating our observations and validating our molecular method.

## Conclusions

Comprehensive NGS evaluation highlights select scenarios in which histologic assessment has limitations and should allow for refinement of the Martini and Melamed criteria for evaluating tumor relatedness. In patients with histopathologically confirmed IM or patients with discordance between the histopathological and mutational evaluations, consideration of our molecular classification can be helpful for differentiation. Overall, our findings suggest that a comprehensive diagnostic approach incorporating histology and molecular analysis is essential to drawing this critical distinction in clinical practice. Molecular staging has the potential to revolutionize the current staging practice in patients with multiple tumors, providing robust confirmation of tumor clonality and information on actionable mutations at the same time.

## Data Availability Statement

The datasets presented in this study can be found in online repositories. The names of the repository/repositories and accession number(s) can be found in the article/**Supplementary Material**. Accession of the submission is: HRA000836 (https://bigd.big.ac.cn/gsa-human/browse/HRA000836.)

## Ethics Statement

The study was approved by the Medical Ethics Committee of Beijing Haidian Hospital (No.2020-041). The patients/participants provided their written informed consent to participate in this study. Written informed consent was obtained from the individual(s) for the publication of any potentially identifiable images or data included in this article.

## Author Contributions

YH and GP contributed to the study design. ML, HW, HC and SC contributed to data bioinformatic analysis and interpretation. YH, GP, XM, QL, DL, YY, SW, and XW contributed to data collection. YH, GP, and ML contributed to the drafting of the article and to its revisions. All authors contributed to the article and approved the submitted version. We thank all the contributing authors for their great effort on this article.

## Conflict of Interest

ML, HC, HW, and SC are employees of Acornmed Biotechnology Co., Ltd.

The remaining authors declare that the research was conducted in the absence of any commercial or financial relationships that could be construed as a potential conflict of interest.

The handling editor declared a shared affiliation, though no other collaboration, with several of the authors GP, XM, QL, DL, YY, SW, XW, and YH.

## Abbreviations

MPLC, multiple primary lung cancer; MLC, multiple lung cancer; sMPLC, synchronous MPLC; mMPLC, metachronous MPLC; CT, computed tomography; NSCLC, non-small-cell lung cancer; IM, intrapulmonary metastasis; NGS, next-generation sequencing; TMB, tumor mutational burden; IAC, invasive adenocarcinoma; AIS, adenocarcinoma *in situ*; MIA, minimally invasive adenocarcinoma; MA, Mucinous adenocarcinoma; GGO, ground-glass opacity; GGN, ground-glass nodule.
