# Supplementary material for: Molecular Identification and Genetic Characterization of Early-Stage Multiple Primary Lung Cancer by Large-Panel Next-Generation Sequencing Analysis
Source: Front Oncol. 2021 May 24;11:653988. doi: 10.3389/fonc.2021.653988 (PMC8183821; doi:10.3389/fonc.2021.653988)
Supplement: Supplementary file 1 [file Presentation_1.pdf]

Supplementary Image

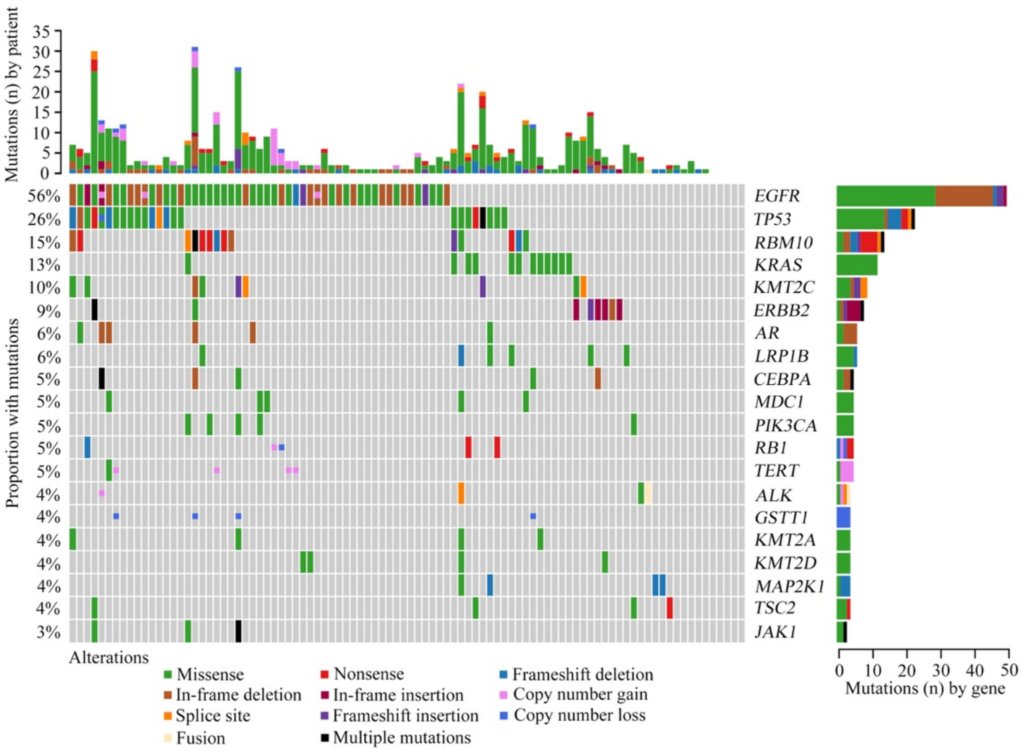

Figure S1. Landscape of somatic mutations in 94 patients with early stage NSCLC.

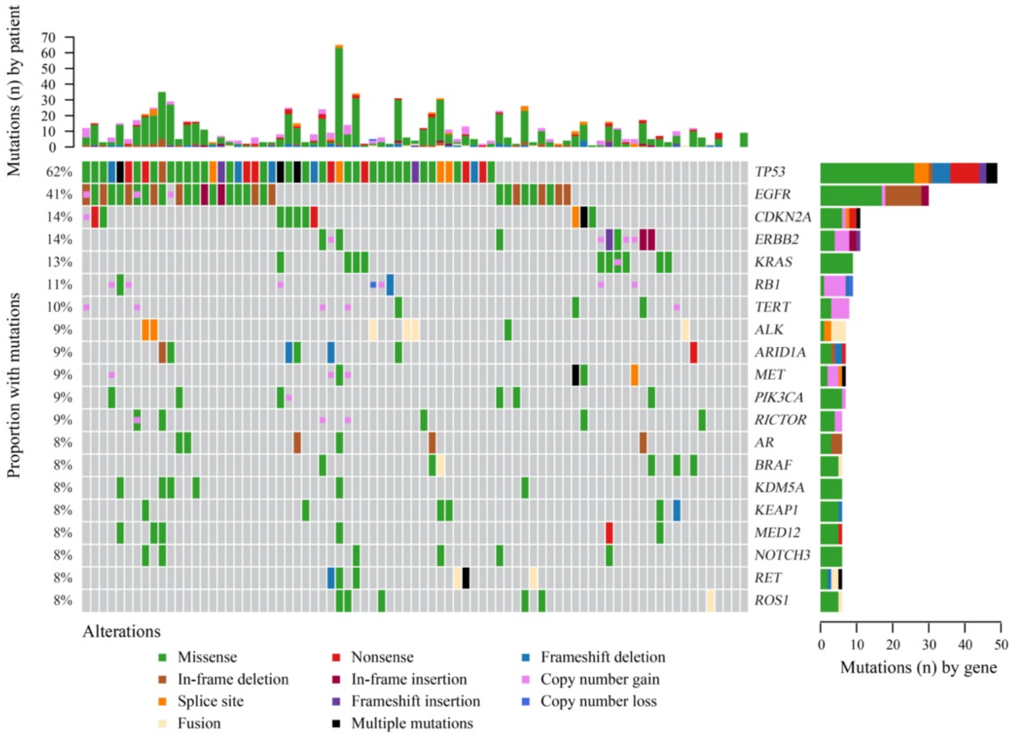

Figure S2. Landscape of somatic mutations in 79 pantients with advanced NSCLC.

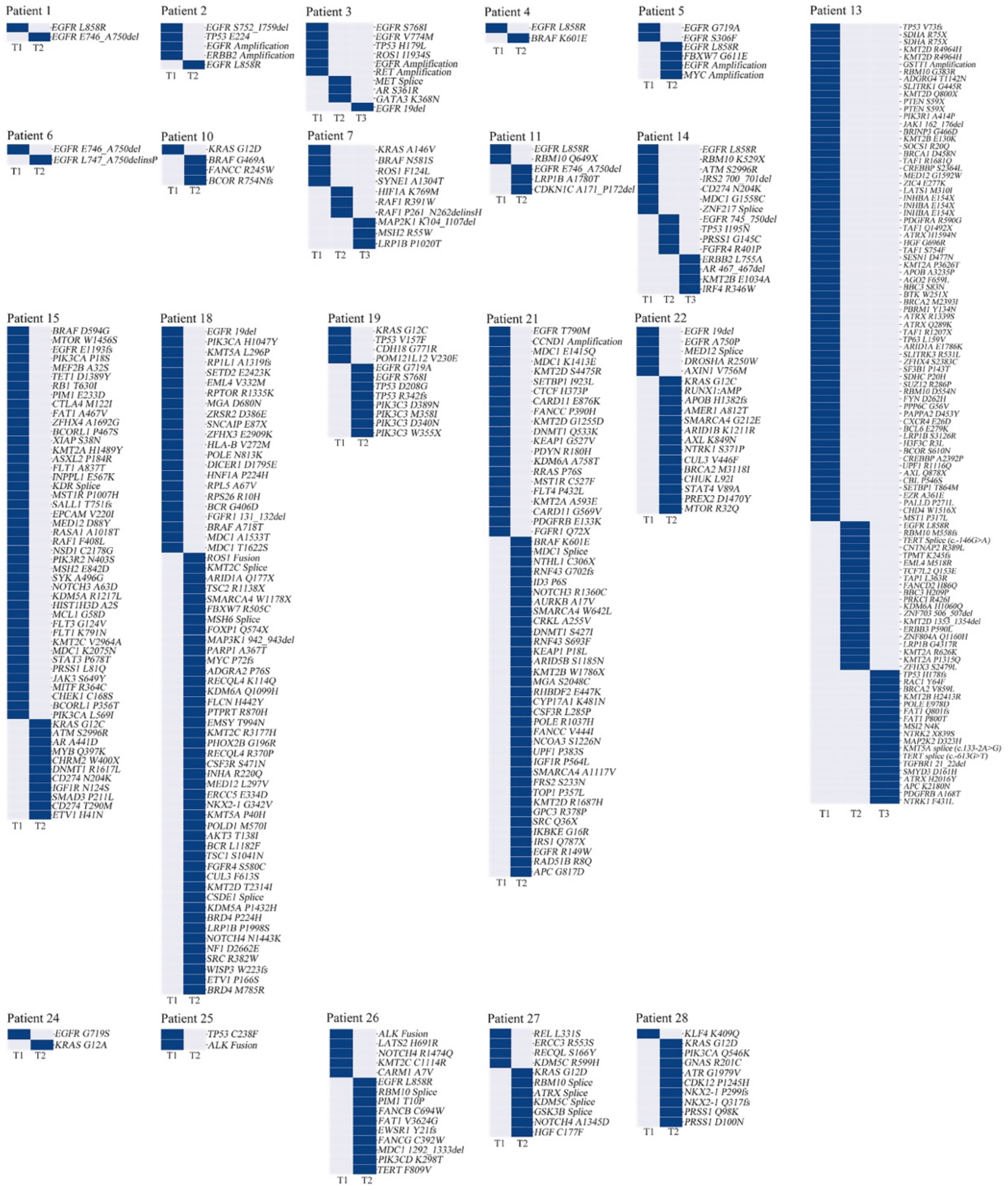

Figure S3. Molecular heatmap of MPLC patients with inconsistent mutations between tumors. The heatmap shows the distribution of all the somatic mutations in 46 tumors from 21 patients. Gray represents the wild type of any genes; blue represents the genomic mutations.

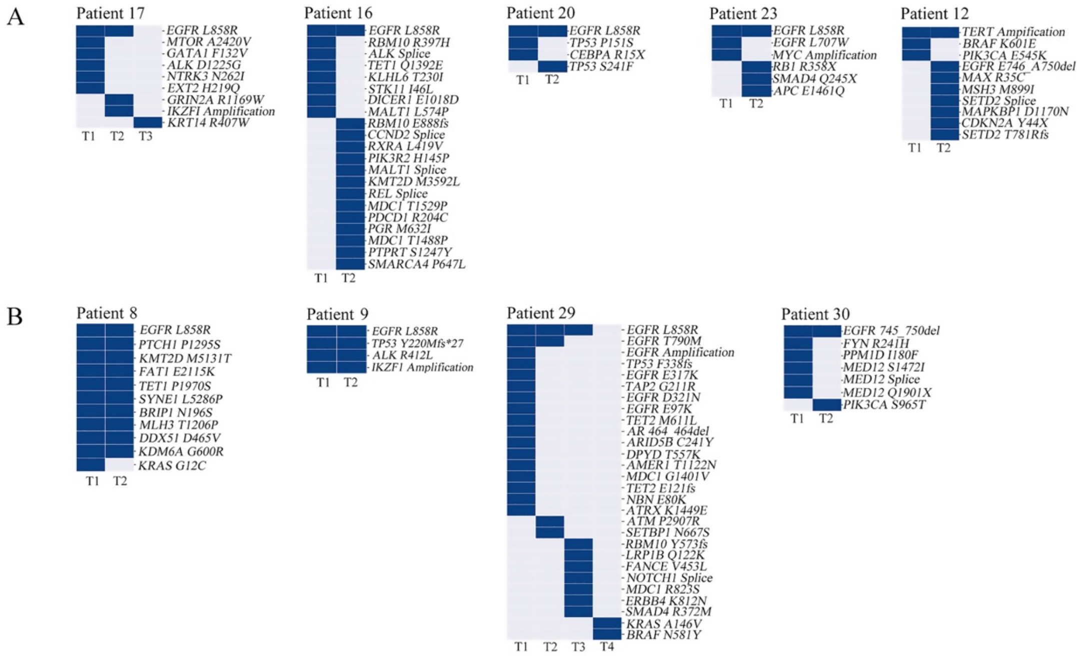

Figure S4. Molecular heatmap of MLC patients with the same mutations between tumors. A, Molecular heatmap of MPLC patients with the identical *EGFR L858R* substitution or *TERT Amplification* between tumors. The heatmap shows the distribution of all the somatic mutations in 11 tumors from 5 patients. B, Molecular heatmap of IM patients with the identical *EGFR L858R* substitution or *exon 19 deletion* between tumors. The heatmap shows the distribution of all the somatic mutations in 10 tumors from 4 patients. Gray represents the wild type of any genes; blue represents the genomic mutations.
