# Supplementary material for: Molecular Identification and Genetic Characterization of Early-Stage Multiple Primary Lung Cancer by Large-Panel Next-Generation Sequencing Analysis
Source: Front Oncol. 2021 May 24;11:653988. doi: 10.3389/fonc.2021.653988 (PMC8183821; doi:10.3389/fonc.2021.653988)
Supplement: Supplementary file 2 [file Table_1.docx]

Supplementary Tables

**Table S1.** Clinical characteristics of 94 Patients with early-stage NSCLC.

| Patient characteristics (N=94) | Number (%) |
| --- | --- |
| Sex, n (%) |  |
| Male | 27 (28.7%) |
| Female | 67 (71.3%) |
| Age(year), y (range) |  |
| Median | 56.5 |
| Range | 34-83 |
| ≤60 | 57 (60.6%) |
| ＞60 | 37 (39.4%) |
| Smoking history, n (%) |  |
| Yes | 25 (26.6%) |
| No | 69 (73.4%) |
| Tumor characteristics (N=94) |  |
| Stage, n (%) |  |
| IA1 | 40 (42.6%) |
| IA2 | 34 (36.2%) |
| IA3 | 20 (21.3%) |
| Histology, n (%) |  |
| AIS | 3 (3.2%) |
| MIA | 17 (18.1%) |
| IAC | 72 (76.6%) |
| SCC | 1 (1.1%) |
| Others | 1 (1.1%) |
| Maximum tumor size, mm (range) |  |
| Median | 12 |
| Range | 1-30 |
| ≤10 | 40 (42.6%) |
| 10-20 | 37 (39.4%) |
| 20-30 | 17 (18.1%) |
| Side, n (%) |  |
| Left | 42 (44.7%) |
| Right | 52 (55.3%) |

AIS: adenocarcinoma in situ; MIA: minimally invasive adenocarcinoma; IAC: invasive adenocarcinoma; SCC: squamous-cell carcinoma.

**Table S2.** Clinical characteristics of 79 patients with advanced-stage NSCLC.

| Patient characteristics (N=79) | Number (%) |
| --- | --- |
| Sex, n (%) |  |
| Male | 48 (60.8%) |
| Female | 31 (39.2%) |
| Age(year), y (range) |  |
| Median | 62 |
| Range | 33-85 |
| ≤60 | 36 (45.6%) |
| ＞60 | 43 (54.4%) |
| Smoking history, n (%) |  |
| Yes | 38 (48.1%) |
| No | 41 (51.9%) |
| Stage, n (%) |  |
| IIIB | 16 (20.2%) |
| IV | 63 (79.8%) |
| Tumor characteristics |  |
| Histology, n (%) |  |
| Adenocarcinoma | 64 (81.0%) |
| Squamous cell carcinomas | 14 (17.7%) |
| Adenosquamous carcinoma | 1 (1.3%) |

**Table S3.** Comparison of clinical characteristics between early-stage NSCLC and advanced-stage NSCLC.

|  | Early-stage | Advanced-stage | P Value |
| --- | --- | --- | --- |
| Patient characteristics | Number (%) | Number (%) |  |
| Sex, n (%) |  |  | ***＜0.0001*** |
| Male | 27 (28.7%) | 48 (60.8%) |  |
| Female | 67 (71.3%) | 31 (39.2%) |  |
| Age(year), y (range) |  |  | 0.1314 |
| Median | 56.5 | 62 |  |
| Range | 34-83 | 33-85 |  |
| ≤60 | 57 (60.6%) | 36 (45.6%) |  |
| ＞60 | 37 (39.4%) | 43 (54.4%) |  |
| Smoking history, n (%) |  |  | ***0.0047*** |
| Yes | 25 (26.6%) | 38 (48.1%) |  |
| No | 69 (73.4%) | 41 (51.9%) |  |
| Tumor characteristics |  |  |  |
| Stage, n (%) |  |  | ***＜0.0001*** |
| AAH | 0 | 0 |  |
| 0 | 0 | 0 |  |
| IA1 | 40 (42.5%) | 0 |  |
| IA2 | 34 (36.2%) | 0 |  |
| IA3 | 20 (21.3%) | 0 |  |
| IB | 0 | 0 |  |
| IIIB | 0 | 16 (20.2%) |  |
| IV | 0 | 63 (79.8%) |  |
| Histology, n (%) |  |  | ***＜0.0001*** |
| AAH | 0 | 0 |  |
| AIS | 3 (3.2%) | 0 |  |
| MIA | 17 (18.1%) | 0 |  |
| IAC | 72 (76.6%) | 64 (81.01%) |  |
| SCC | 1 (1.1%) | 14 (17.72%) |  |
| ASC | 0 | 1 (1.27%) |  |
| Others | 1 (1.1%) | 0 |  |

AAH: atypical adenomatous hyperplasia; AIS: adenocarcinoma in situ; MIA: minimally invasive adenocarcinoma; IAC: invasive adenocarcinoma; SCC: squamous-cell carcinoma; ASC: Adenosquamous carcinoma.
